# Meningitis caused by *Candida dubliniensis* in a patient with meningioma: a systematic review of the literature

**DOI:** 10.1007/s11845-026-04433-9

**Published:** 2026-05-15

**Authors:** Nurgül Samancı, Elif Ayça Şahin, Yesim Yildiz, Emrah Çeltikçi, Muammer Melih Şahin, Derya Göksu Fidan, Ayşe Kalkancı, Kayhan Çağlar

**Affiliations:** 1 Faculty of Medicine, Department of Medical Microbiology, Gazi University, Ankara, Türkiye; 2 Faculty of Medicine, Department of Infectious Disease and Clinical Microbiology, Gazi University, Ankara, Türkiye; 3 Faculty of Medicine, Department of Neurosurgery, Gazi University, Ankara, Türkiye; 4 Faculty of Medicine, Department of Otorhinolaryngology, Gazi University, Ankara, Türkiye; 5 School of Medicine, Koc University, Istanbul, Türkiye

**Keywords:** *Candida dubliniensis*, *Candida* meningitis, Clival meningioma, Systematic review

## Abstract

**Background:**

*Candida* meningitis is a rare but life-threatening infection, most commonly caused by *Candida albicans*. *Candida dubliniensis*, despite its close phenotypic similarity to *C. albicans*, is an uncommon cause of central nervous system (CNS) infections, with limited data available. To date, only 12 cases of *C. dubliniensis* meningitis have been reported worldwide. This study presents the 13th documented case and the first reported from Türkiye, together with a systematic review of the literature.

**Case presentation:**

A 44-year-old immunocompetent male developed meningitis caused by *C. dubliniensis* following subtotal resection of a clival meningioma. The diagnosis was established through fungal culture, MALDI-TOF MS, and ITS region DNA sequencing. The isolate was deposited in GenBank (accession number PV088619). Antifungal therapy with fluconazole was initiated based on susceptibility results. Despite appropriate antifungal management, the patient developed secondary *Klebsiella pneumoniae* sepsis and subsequently died, highlighting the complexity and high mortality risk associated with post-neurosurgical fungal CNS infections.

**Methods:**

A systematic review was conducted according to PRISMA guidelines using PubMed and Web of Science databases, including studies published up to February 2025. Search terms included “*Candida dubliniensis*” and “meningitis.” Only microbiologically confirmed cases with individual patient data were included. Studies older than 25 years or lacking sufficient diagnostic detail were excluded.

**Results and conclusion:**

Thirteen cases were identified. Most patients were immunocompetent, and infections were frequently acquired outside neurosurgical settings. Mortality was reported in four cases. These findings emphasize the extreme rarity of *C. dubliniensis* meningitis and underscore the importance of early species-level identification strategies.

## Introduction

*Candida dubliniensis* is a yeast first identified in 1995 in the oral cavity of HIV-infected patients in Dublin, Republic of Ireland [1]. Since its discovery, *C. dubliniensis* has gained increasing attention, although it is less prevalent and less virulent than *C. albicans*. Its identification can be challenging due to phenotypic similarities with *C. albicans*, which may delay appropriate treatment. Despite nearly three decades since its discovery, many aspects of *C. dubliniensis* remain unclear [2].

*Candida* meningitis is a rare condition, most commonly associated with *C. albicans*, with risk factors including recent abdominal surgery, broad-spectrum antibiotic therapy, indwelling catheters, and immunocompromised states such as steroid use and malignancy [3, 4]. However, *Candida* meningitis following neurosurgical procedures is uncommon. In a cohort of 400 patients over a 5-year period, only 3 cases of postsurgical *Candida* meningitis were reported [5].

In this report, we describe a patient with meningioma who developed postsurgical C. dubliniensis meningitis. We also performed a systematic literature review focusing on risk factors, diagnosis, treatment, and outcomes. To date, only 12 cases of C. dubliniensis meningitis have been reported worldwide [6–17]. This study presents the first case from Türkiye and the 13th reported case globally.

In this case report, we describe a case with meningioma who developed postsurgical *C. dubliniensis* meningitis. We performed a systematic literature review with special focus on common risk factors, diagnosis, treatment and survival. Only 12 cases of *C. dubliniensis* meningitis have been reported worldwide [6–17]. This article presents the first case in Türkiye and the 13th published case globally. The case was further complicated by clival meningioma and septic shock caused by *Klebsiella pneumoniae*, making it particularly challenging.

### Case report

**Fig. 1:**
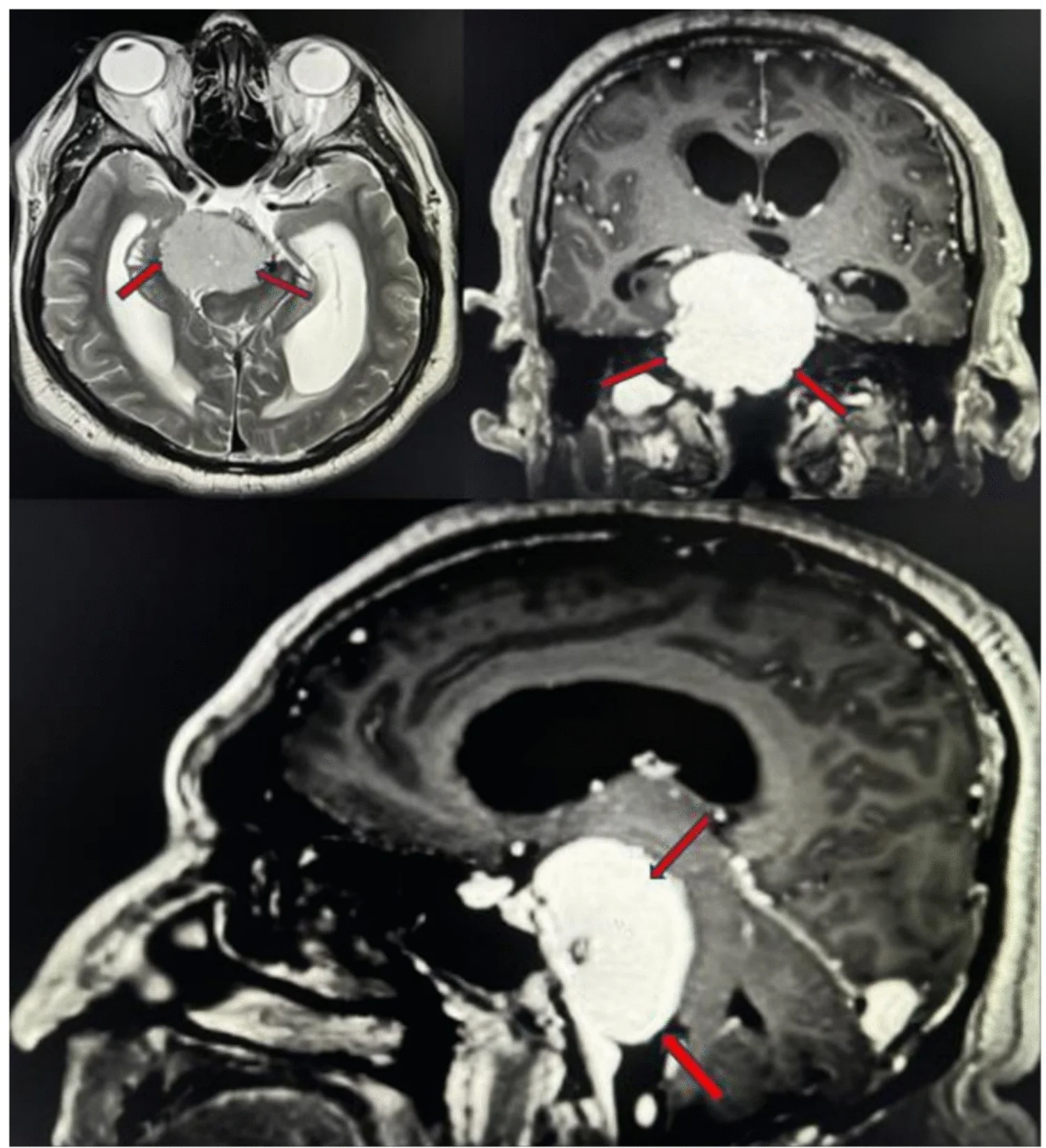
Top left T1 sequence sagittal view of the mass, top right coronal view of the mass, bottom axial view of the mass (red arrows)

A 44-year-old male presented to the neurology outpatient clinic on November 18th, 2024 with a five-month history of numbness in both hands, feet, and his face. Cranial magnetic resonance imaging (MRI) revealed a clival meningioma (Fig. 1), and he was subsequently referred to the neurosurgery department on November 18th, 2024. His symptoms included numbness in all four extremities, predominantly on the right side, right-sided facial numbness, and loss of taste sensation. His medical history included glaucoma, for which he was under follow-up. He had no history of surgery, smoking, or alcohol consumption and was unemployed at the time of admission. The patient was admitted to the neurosurgery ward for surgical intervention. Cranial Magnetic Resonance Imaging (MRI) and Computed Tomography (CT) were performed for localization. No signs of increased intracranial pressure (ICP) were detected. Cranial MRI revealed a well-defined extraaxial mass measuring 41 × 27 × 50 mm, with diffuse homogeneous contrast enhancement following intravenous contrast administration. The lesion had a broad dural base and exerted severe compression on the right temporal lobe, mesial temporal gyrus, right half of the mesencephalon, pons, and medulla at the level of the right pontocerebellar angle. The mass was suspected to originate from the dorsum sella-clival dura. Figure 1 shows pre-op MRI of the patient. Cranial CT showed a mass lesion approximately 35 × 45 mm in the prepontine area, compressing the pons and mesencephalon while extending anteriorly into the right cavernous sinus. Calcifications were observed in the right half of the lesion. The patient underwent subtotal resection of the clival mass extending to the brainstem via an endoscopic endonasal transsphenoidal approach.

On postoperative day 17, the patient underwent endoscopic repair of cerebrospinal fluid (CSF) rhinorrhea, and a lumbar drainage catheter was placed. On postoperative day 19, due to a suspected infection, a lumbar puncture (LP) was performed. CSF analysis revealed clear material, elevated protein 1534 mg/dL level, and a decreased glucose 105 mg/dL. No microorganisms were detected by direct examination and Gram staining. However, yeast colonies were isolated from CSF culture after 24 h of incubation. The organism was identified as *Candida dubliniensis* at the species level using Matrix Assisted Laser Desorption Ionization-Time of Flight Mass Spectrometry (MALDI-TOF MS, Vitek MS, bioMérieux, France). Laboratory results at that time were as follows: C-reactive protein (CRP) 29 mg/dL, white blood cell count (WBC) 9,950/mm^3^. Tests for hepatitis B virus (HBV), hepatitis C virus (HCV), and human immunodeficiency virus (HIV) were negative.

### Mycological analysis and follow up the patient

Identification of *C. dubliniensis* by MALDI-TOF MS was confirmed using multiple complementary methods, including germ tube testing, evaluation of chlamydospore formation on cornmeal Tween 80 agar, assessment of growth at 42 °C, and carbohydrate assimilation profiling using the API ID32C system (bioMérieux, France). Additionally, the morphological appearance of CHROMagar Plus was examined (CHROMagarTM) and Internal Transcribed Spacer (ITS) region of fungal ribosomal DNA (rDNA) was sequenced. Genomic DNA was extracted from yeast colonies using the EurX GeneMATRIX Plant & Fungi DNA Isolation Kit (Poland). Polymerase Chain Reaction (PCR) amplification targeted the ITS region of fungal rDNA using the ITS1 (5′-TCCGTAGGTGAACCTGCGG-3′) and ITS4 (5′-TCCTCCGCTTATTGATATATGC-3′) primers, which amplify the ITS1 region and the 5.8S rRNA gene. A one-step PCR procedure was performed to amplify a region of approximately 700 base pairs using the FIREPol® DNA Polymerase (Solis Biodyne, Estonia). Following PCR, a single band was observed on an agarose gel, confirming successful amplification. For PCR product purification, samples showing a single band were purified using the HighPrep™ PCR Clean-up System (MAGBIO, AC-60005), following the manufacturer’s protocol. Sanger sequencing was conducted using the ABI 3730XL Sanger sequencing instrument and the BigDye Terminator v3.1 Cycle Sequencing Kit (Applied Biosystems, Foster City, CA). The sequencing reads obtained with the ITS1 and ITS4 primers were assembled into contigs to generate a consensus sequence using the CAP contig assembly algorithm within BioEdit software. The obtained sequence was submitted to GenBank under accession number PV088619. A BLAST search in the NCBI PubMed DNA database identified the sequence as 100% identical to *Candida dubliniensis*.

**Fig. 2:**
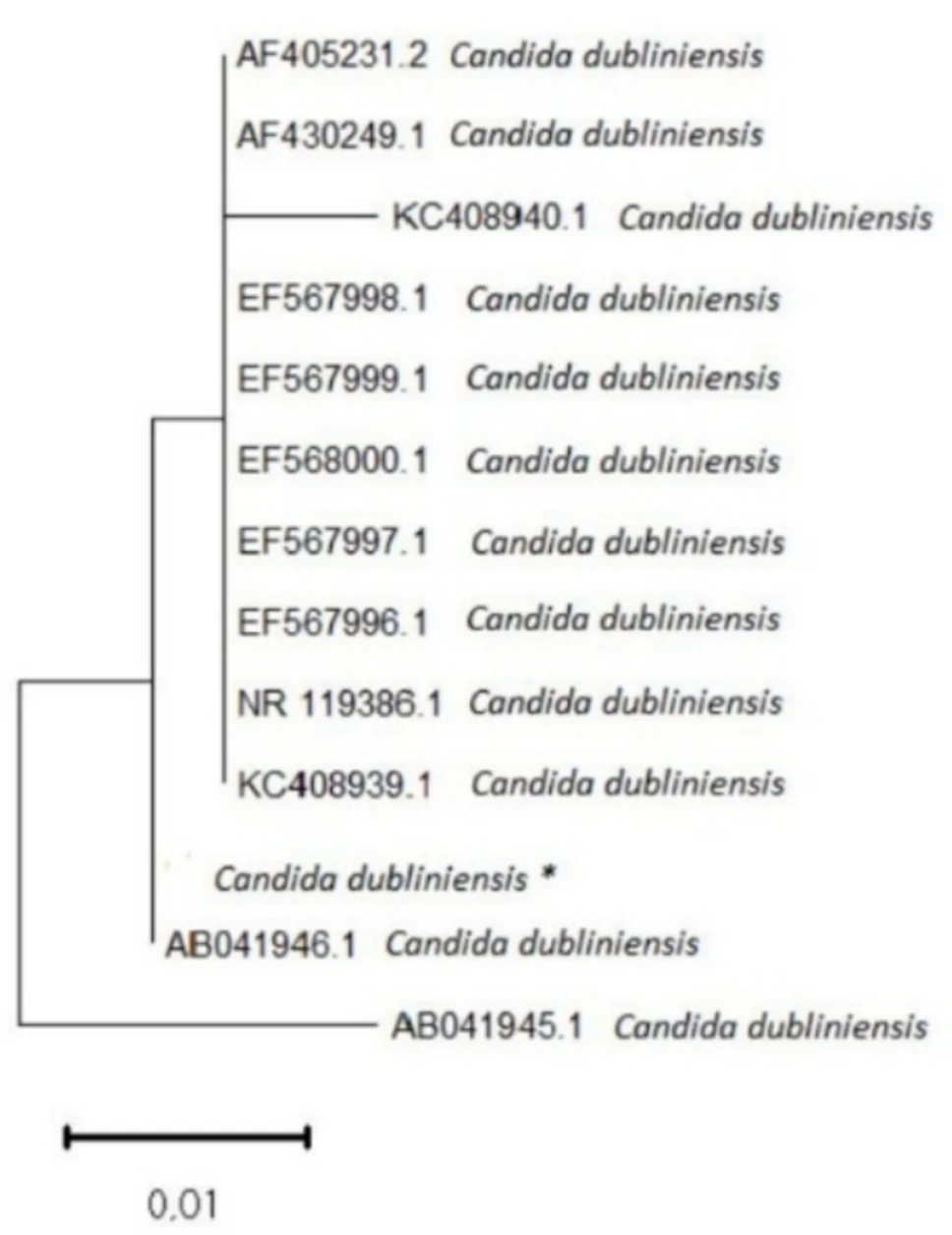
Phylogenetic tree of our *Candida dubliniensis* strain (PV088619.1)

The evolutionary history was inferred using the Maximum Likelihood method based on the Tamura–Nei model [18]. The tree with the highest log likelihood (−6416,94) is shown in Fig. 2. Initial tree(s) for the heuristic search were obtained automatically by applying Neighbor-Join and BioNJ algorithms to a matrix of pairwise distances estimated using the Tamura-Nei model, and then selecting the topology with superior log likelihood value. The tree is drawn to scale, with branch lengths measured in the number of substitutions per site. This analysis involved 13 nucleotide sequences. There were a total of 4622 positions in the final dataset. Evolutionary analyses were conducted in MEGA11 [19].

Minimum inhibitory concentrations (MICs) were determined using the broth microdilution method following Clinical and Laboratory Standards Institute (CLSI) guidelines M27-A3/S4 in 96-well plates [20]. The tested antifungal agents included amphotericin B (AMB), fluconazole (FLC), itraconazole (ITC), voriconazole (VRC), and caspofungin (CAS). The MIC values were 0.25 μg/L for fluconazole (susceptible), 0.125 μg/L for amphotericin B (susceptible), 0.125 μg/L for voriconazole (susceptible) and 0.125 μg/L for itraconazole (susceptible). Based on the results of the antifungal susceptibility test, fluconazole treatment was initiated. No growth was detected in simultaneously collected blood and urine cultures. However, CSF cultures performed on the 2nd and 4th days of antifungal therapy showed persistent growth of *Candida dubliniensis*. On the 34th postoperative day, hospital-acquired *Klebsiella pneumoniae* was isolated from blood, endotracheal aspirate, and CSF cultures. Colistin was added to the treatment regimen based on antibiogram results. The patient was admitted to the intensive care unit (ICU) due to a Glasgow Coma Scale (GCS) score of III. Histopathological examination confirmed the diagnosis of secretory meningioma, WHO Grade 1, located in the clivus. Immunohistochemical analysis showed tumor cells positive for SSTR2A and EMA, while negative for S-100. Despite treatment, the patient died with septic shock secondary to meningitis on the 44th day of surgical operation, 25th day of antifungal therapy and the eighteenth day of colistin treatment.

As the decision to publish the case was made after the patient’s death, retrospective ethical approval was obtained from the Non-Interventional Research Ethics Committee of Gazi University (Decision No: 061, dated April 18, 2025). Informed consent was obtained from a first-degree relative of the patient.

### Systematic review of the literature

**Fig. 3:**
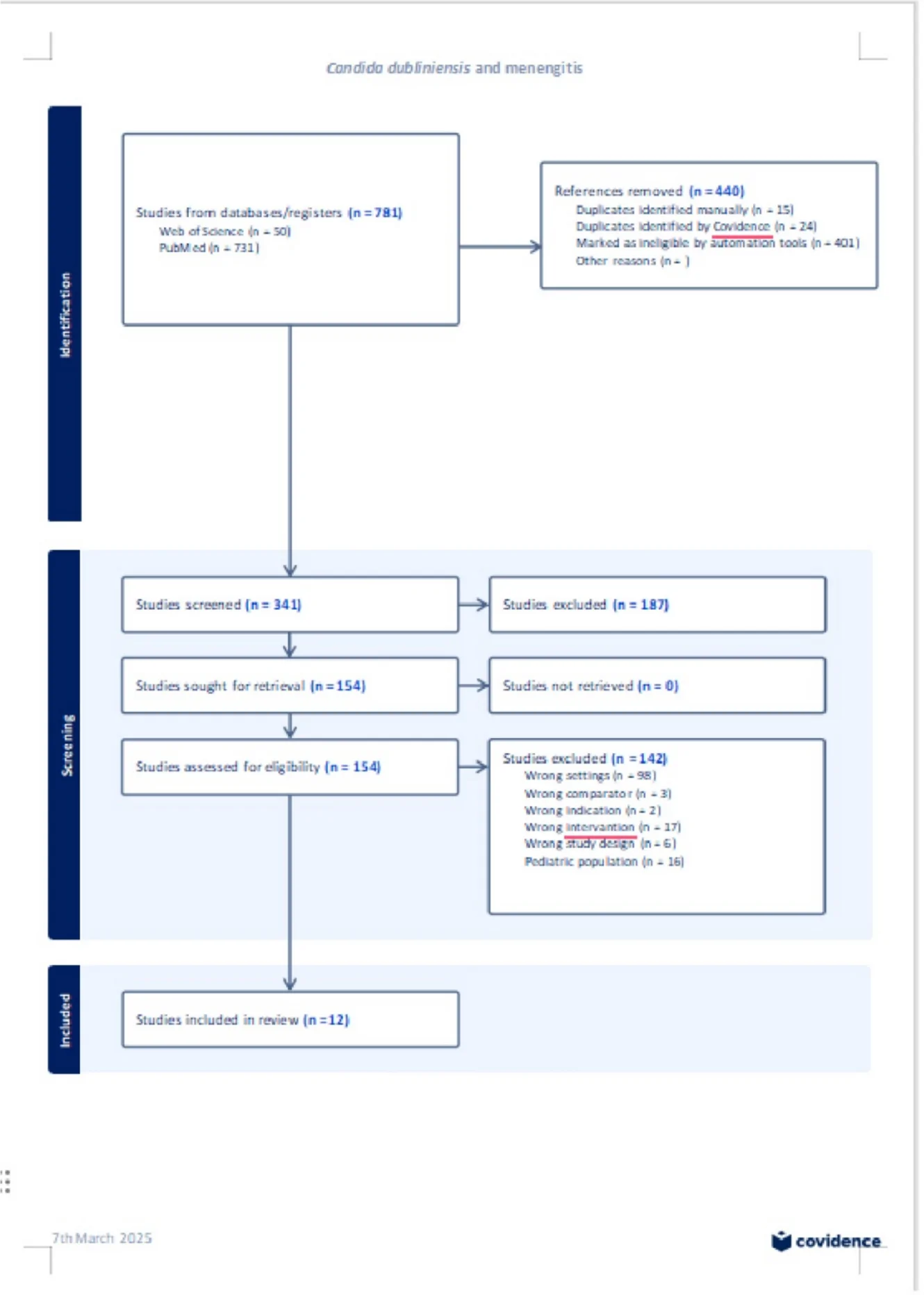
PRISMA flow diagram of the systematic literature search and screening

Literature Review and Study Selection PubMed and Web of Science databases were queried from their inception until February 2025 using the following combination of keywords: (*Candida*) AND (Meningitis) AND (*Candida dubliniensis*). Case reports of patients diagnosed with *Candida* meningitis were screened. Reference lists of relevant articles were manually searched to identify additional qualifying articles not included in the original database search. Studies of patients who were not diagnosed with *Candida* meningitis were excluded. Patients whose lesion was not determined to be fungal by Gram stain, CSF culture, polymerase chain reaction test, or other laboratory or pathologic tests were excluded. Articles without abstract or full-text available in English or did not include original patient data (e.g., review articles, commentaries, letters) were excluded. Articles published more than 25 years ago were excluded. Screening of records was performed by two blind reviewers following the Preferred Reporting Items for Systematic reviews and Meta-Analyses (PRISMA) protocol (Fig. 3). All conflicts were resolved by a third reviewer under the guidance of the senior author (A.K.)

The database search identified 781 articles (PubMed: 731, WOS: 50). There were 15 duplicates identified manually, 24 duplicates identified by Covidence, 401 references marked as ineligible by automation tools. 341 studies screened against title and abstract. 187 studies excluded, 154 studies assessed for full-text eligibility, 142 studies excluded for 98 wrong setting, 17 wrong intervention, 16 pediatric population, 6 wrong study design, 3 wrong comparator, 2 wrong indication. A total of 12 case reports describing *C. dubliniensis* meningitis were included in the analysis (Fig. 3).

A total of 13 patients, including the present case, were identified over the past 25 years [6–17]. The majority of those patients were from two countries United States (n:4) and Australia (n:4), with 4 of them originating from other countries (Saudi Arabia, Denmark, Canada, Ireland). The included cohort consists of 12 cases, but age data is available for 7 cases. Based on these known ages, between 22—70, the average age is 38.9 years, and the median age is 32 years. Gender distribution could not be determined due to missing data. 3 cases were female, 3 case were male. In 4 out of the 8 cases, were immunocompetant. None of the cases was surgery related. Details of the included studies characteristics are summarized in Table 1. CSF cultures after lumbar puncture were performed in all cases, two were failed. In this two cases fungal PCR, beta glucan testing and sequencing were performed. All patients received antifungal medical treatment, most of them included Amphotericin B. Patient outcome was reported in all cases. Four patients, including our case, died, whereas the remaining nine recovered. DNA identification was performed for 4 out of 13 cases including ours.

**Table 1:** Published cases of *Candida dubliniensis* according to literature (PRISMA)

Ref no	Citation	Underlying Disease	Risk factor	Diagnosis Method	Treatment	Outcome	DNA profile
6	Gheisghahi M, et al. Med Mycol Case Rep. 2019; 27: 22—24	Liver transplant	Immunsupression	CSF culture	Not mentioned	Died	No
7	Wilson MR, et al. JAMA Neurol. 2018; 75 (8): 947—955	None	None	Metagenomic Next-Generation Sequencing	Not mentioned	Recovered	Yes
8	Van Hal SJ, et al. Emerg Infect Dis. 2008; 14(2): 327—329	Heart–Lung transplant	Immunsupression	CSF culture	Not mentioned	Recovered	No
9	Andrew NH, et al. BMJ Case Rep. 2011; 2011:bcr0620114384	None	None	CSF culture	Liposomal amphotericin B, fluconazole	Recovered	No
10	Tahir M, et al Front Neurol. 2020; 11: 601242	Hepatitis C, Cirrhosis		Multiple lumbar punctures, CSF fungal culture	Liposomal amphotericin B, fluconazole	Recovered	No
11	Yamahiro A, et al. Mycopathologia. 2016; 181 (7—8): 589—592	Cirrhosis	IV drug use	CSF culture	Liposomal amphotericin B, flucytosine	Died	No
12	Price C. et l. IDCases. 2022; 31: e01665	None	IV drug use	CSF culture	Not mentioned	Recovered	No
13	Absar M, et al. Med Mycol Case Rep. 2024; 46: 100678	Cirrhosis	Multiple comorbidities	CSF culture	Liposomal amphotericin B, anidulafungin	Recovered	No
14	Babici D, et al. eNeurologicalSci. 2024; 36: 100519	Viral myocarditis & Takutsobo cardiomyopathy	None	CSF culture	Amphotericin B, flucytosine	Recovered	No
15	Herrera S, et al. Med Mycol Case Rep. 2019; 24: 41—43	Lung transplant	Immunsupression	Fungal PCR, BD glucan assay	Not mentioned	Recovered	Yes
16	Kelly L, et al. Br. J Neurosurgery. 2023; 37 (2): 227—230	Meningitis/ventriculitis	CNS device	CSF culture, 18 s rRNA testing	Liposomal amphotericin B, fluconazole, flucytosine	Died	Yes
17	Askari A, et al. Neurol Clin Pract. 2024; 14 (4): e200279	None	Opioid use disorder	CSF culture, posterior fossa arachnoid sample	Fluconazole	Recovered	No
	**OUR CASE**	**Meningioma**	**Transsphenoidal resection Surgery**	**CSF culture**	**Fluconazole**	**Died**	**Yes**

## Discussion

More than 90% of invasive *Candida* infections are caused by *Candida albicans*, *Nakaseomyces glabrata* (previously *Candida*), *Candida parapsilosis*, and *Pichia kudriavzevii* (previously *Candida*). Central nervous system (CNS) infections caused by *Candida* in immunocompromised patients are rare but have a mortality rate exceeding 50% [21]. *Candida* meningitis, primarily affects immunocompromised individuals, including neonates, organ transplant recipients, and patients with prolonged hospitalization. Neurosurgical interventions may increase the risk of infection. [3, 4]. Here, we report a surgery-related meningitis case caused by *C. dubliniensis*. In our case, prior neurosurgical intervention and the presence of a clival meningioma likely contributed to the development of CNS infection. This represents the first reported case in Türkiye and the 13th documented case worldwide. Table 1 provides a summary of all reported 13 cases of *C. dubliniensis* meningitis.

Bridges et al^5^ reported 3 cases of *Candida* meningitis after endoscopic transnasal approach for transsphenoidal surgery, same surgical procedure with our case. Risk factors were similar to our case, laboratory values are not consistently helpful for diagnosis as we assumed.

*Candida dubliniensis* was first isolated from a meningitis case in 2008 [8]. This species is closely related to *C. albicans* and shares similar characteristics, including hyphae and chlamydospore formation [22]. Researchers predominantly isolated *Candida dubliniensis* from the oral cavities of HIV and AIDS patients, though it was also found at other body sites. Although *Candida* species are frequently isolated from mucosal surfaces, CNS involvement is rare. Risk factors for CNS candidiasis include malignancy, solid tumors, organ transplantation, intravenous drug use, diabetes, and HIV infection. Additionally, the presence of intracranial devices and prior neurological procedures further predispose individuals to infection [23].

Diagnosis of *Candida* meningitis is challenging due to its nonspecific clinical presentation, including fever, headache, altered mental status, and seizures. Cerebrospinal fluid (CSF) analysis typically reveals elevated protein levels, low glucose, and lymphocytic pleocytosis. Microbiological confirmation is achieved through CSF culture, but sensitivity is low. Biomarkers like (1,3)-β-D-glucan and CSF PCR for *Candida* species improve diagnostic accuracy. Liposomal amphotericin B with or without flucytosine is the first-line treatment. Fluconazole may also be used in less severe cases or as step-down therapy. The duration of treatment is typically prolonged, often lasting several weeks. Prognosis depends on early diagnosis and treatment initiation. Mortality remains high, especially in neonates and severely immunocompromised patients. Long-term neurological complications are common. Early recognition and appropriate antifungal therapy are crucial in managing *Candida* meningitis and improving patient outcomes [3, 4].

Concluding remarks of our case report: *Candida dubliniensis* is a rare cause of meningitis but should be considered in patients with neurosurgical history and persistent signs of CNS infection. Early diagnosis and prompt initiation of antifungal therapy are critical for improving outcomes. Neurosurgical interventions and CSF leaks are potential risk factors for fungal meningitis, emphasizing the need for rigorous infection control and monitoring in post-neurosurgical patients. Despite appropriate antifungal therapy, persistent CSF culture positivity may indicate treatment failure, requiring alternative antifungal strategies. Hospital-acquired bacterial infections can complicate fungal infections, increasing mortality risk. Multidisciplinary management involving neurosurgeons, infectious disease specialists, and microbiologists is essential for improving patient outcomes in fungal CNS infections.

## Data Availability

This manuscript does not report data generation or analysis.

## Abbreviations

CSF: Cerebrospinal fluid
CNS: Central nervous system
MRI: Magnetic resonance imaging
CT: Computed tomography
IIP: Increased intracranial pressure
LP: Lumbar puncture
ICU: Intensive care unit
GCS: Glasgow coma scale
MALDI-TOF: Matrix assisted laser desorption ionization-time of flight mass spectrometry
MIC: Minimum inhibitory concentrations
CLSI: Clinical and laboratory standards institute
AMB: Amphotericin B
FLC: Fluconazole
ITC: Itraconazole
VRC: Voriconazole
CAS: Caspofungin

## References

[1] Sullivan DJ, Westerneng TJ, Haynes KA et al (1995) *Candida dubliniensis* sp nov.: phenotypic and molecular characterization of a novel species associated with oral candidosis in HIV-infected individuals. Microbiology (Reading) 141(Pt 7):1507–1521. 10.1099/13500872-141-7-1507 7551019

[2] Gómez-Gaviria M, Baruch-Martínez DA, Mora-Montes HM (2024) Exploring the biology, virulence, and general aspects of *Candida dubliniensis*. Infect Drug Resist 17:5755–5773. 10.2147/IDR.S497862 PMC11669290 39722735

[3] Góralska K, Blaszkowska J, Dzikowiec M (2018) Neuroinfections caused by fungi. Infection 46(4):443–459. 10.1007/s15010-018-1152-2 PMC6096918 29785613

[4] Nathan CL, Emmert BE, Nelson E, Berger JR (2021) CNS fungal infections: a review. J Neurol Sci 422:117325. 10.1016/j.jns.2021.117325 33516057

[5] Bridges KJ, Li R, Fleseriu M, Cetas JS (2017) *Candida* meningitis after transsphenoidal surgery: a single-institution case series and literature review. World Neurosurg 108:41–49. 10.1016/j.wneu.2017.08.115 28847554

[6] Gheshlaghi M, Helweg-Larsen J (2019) Fatal chronic meningitis caused by *Candida dubliniensis* after liver transplantation. Med Mycol Case Rep 27:22–24. 10.1016/j.mmcr.2019.12.009 PMC6926290 31890490

[7] Wilson MR, O’Donovan BD, Gelfand JM, Sample HA, Chow FC, Betjemann JP, Shah MP, Richie MB, Gorman MP, Hajj-Ali RA, Calabrese LH, Zorn KC, Chow ED, Greenlee JE, Blum JH, Green G, Khan LM, Banerji D, Langelier C, Bryson-Cahn C, Harrington W, Lingappa JR, Shanbhag NM, Green AJ, Brew BJ, Soldatos A, Strnad L, Doernberg SB, Jay CA, Douglas V, Josephson SA, DeRisi JL (2018) Chronic meningitis investigated via metagenomic next-generation sequencing. JAMA Neurol 75(8):947–955. 10.1001/jamaneurol.2018.0463 PMC5933460 29710329

[8] van Hal SJ, Stark D, Harkness J, Marriott D (2008) *Candida dubliniensis* meningitis as delayed sequela of treated *C. dubliniensis* fungemia. Emerg Infect Dis 14(2):327–329. 10.3201/eid1402.070985 PMC2600202 18258133

[9] Andrew NH, Ruberu RP, Gabb G (2011) The first documented case of *Candida dubliniensis* leptomeningeal disease in an immunocompetent host. BMJ Case Rep 2011:bcr0620114384. 10.1136/bcr.06.2011.4384 PMC4545036 22687683

[10] Tahir M, Peseski AM, Jordan SJ (2020) Case report: *Candida dubliniensis* as a cause of chronic meningitis. Front Neurol 11:601242. 10.3389/fneur.2020.601242 PMC7752766 33363510

[11] Yamahiro A, Lau KH, Peaper DR, Villanueva M (2016) Meningitis caused by *Candida dubliniensis* in a patient with cirrhosis: a case report and review of the literature. Mycopathologia 181(7–8):589–593. 10.1007/s11046-016-0006-7 27038312

[12] Price C, Wilson I, Catchpoole E (2023) *Candida dubliniensis* chronic meningitis in an immunocompetent patient: case report and literature review. IDCases 31:e01665. 10.1016/j.idcr.2022.e01665 PMC9801098 36589766

[13] Absar M, Alduwayriji A, Al-Arfaji A, Alduwayrij A, Al-Arfaj A, Shah Z, Nashmy F, Yacoubi MT (2024) Meningitis caused by *Candida dubliniensis* in a patient with liver cirrhosis: a case report and review of the literature. Med Mycol Case Rep 46:100678. 10.1016/j.mmcr.2024.100678 PMC11532277 39497684

[14] Babici D, Mohamed AA, Mattner O et al (2024) *Candida dubliniensis* meningitis in an immunocompetent patient: A case report and review of the literature. eNeurologicalSci 36:100519. 10.1016/j.ensci.2024.100519 PMC11344001 39185332

[15] Herrera S, Pavone P, Kumar D et al (2019) Chronic *Candida dubliniensis* meningitis in a lung transplant recipient. Med Mycol Case Rep 24:41–43. 10.1016/j.mmcr.2019.03.004 PMC6439223 30976504

[16] Kelly L, Walsh J, Skally M et al (2023) *Candida* meningitis/ventriculitis over a decade. Increased morbidity and length of stay a concern. Br J Neurosurg 37(2):227–230. 10.1080/02688697.2022.2054947 35361033

[17] Askari A, Benson J, Felipe Bastos Horta L et al (2024) *Candida dublinensis* meningitis in an immunocompetent host: a case report and review of the literature. Neurol Clin Pract 14(4):e200279. 10.1212/CPJ.0000000000200279 PMC11129330 38808026

[18] Tamura K, Nei M (1993) Estimation of the number of nucleotide substitutions in the control region of mitochondrial DNA in humans and chimpanzees. Mol Biol Evol 10:512–526. 10.1093/oxfordjournals.molbev.a040023 8336541

[19] Tamura K, Stecher G, Kumar S (2021) MEGA11: molecular evolutionary genetics analysis version 11. Mol Biol Evol 38(7):3022–3027. 10.1093/molbev/msab120 PMC8233496 33892491

[20] Clinical and Laboratory Standards Institute. Reference method for broth dilution antifungal susceptibility testing of yeasts,. 2nd ed., Clinical and Laboratory Standards Institute, Wayne PA; 2008. [Approved standard M27-A3/S4]

[21] Denning DW (2024) Global incidence and mortality of severe fungal disease. Lancet Infect Dis 24(7):e428–e438. 10.1016/S1473-3099(23)00692-8 38224705

[22] Moran GP, Coleman DC, Sullivan DJ (2012) *Candida albicans* versus *Candida dubliniensis*: why is *C. albicans* more pathogenic? Int J Microbiol 2012:205921. 10.1155/2012/205921 PMC3166774 21904553

[23] Coulibaly NJ, Fassina GR, Donkor EO, Spence CA (2024) Cervical intradural fungal infection masquerading as a meningioma in an immunocompetent patient: a case report and systematic review. World Neurosurg 192:36–42. 10.1016/j.wneu.2024.08.150 39242026

